# Interrelationship between optic disc edema, spontaneous venous pulsation and intracranial pressure

**DOI:** 10.4103/0301-4738.55061

**Published:** 2009

**Authors:** Nikhil S Choudhari, Rajiv Raman, Ronnie George

**Affiliations:** Department of Glaucoma and Neuro-ophthalmology, Medical Research Foundation, Sankara Nethralaya, 18/41, College Road, Chennai - 600 006, India; 1 Department of Vitreo-retina, Medical Research Foundation, Sankara Nethralaya, 18/41, College Road, Chennai - 600 006, India; 2 Department of Glaucoma, Medical Research Foundation, Sankara Nethralaya, 18/41, College Road, Chennai - 600 006, India

Dear Editor,

Spontaneous venous pulsation (SVP) is a result of the variation in the pressure gradient along the retinal vein as it traverses the lamina cribrosa.[1] When the intracranial pressure (ICP) rises, the intracranial pulse pressure also rises to equal the intraocular pulse pressure and the SVP ceases.

**Figure 1:**
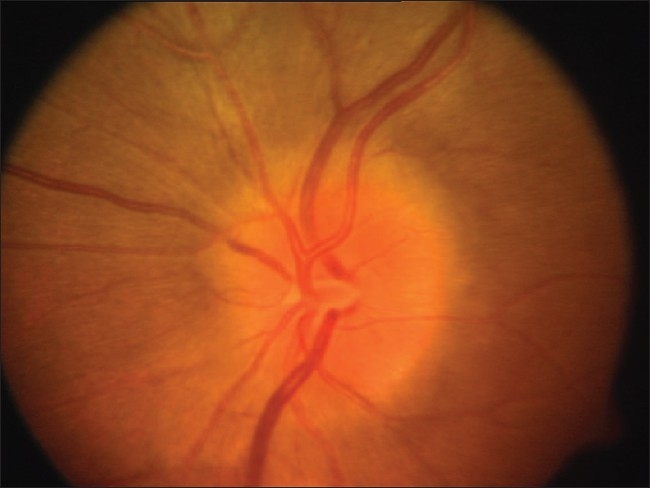
Photograph of the Left optic disc showing disc edema

**Figure 2:**
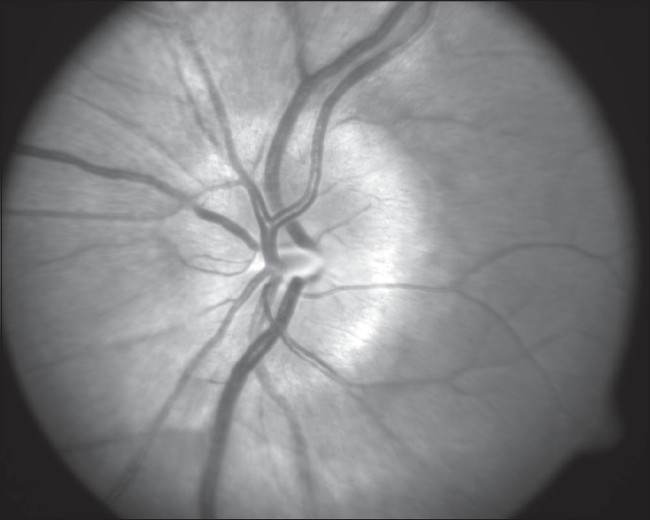
As she is one eyed and papilledema is an emergency, we obtained fundus photographs after she received treatment for raised intracranial pressure for 5 days. The visible optic disc edema is less than that was seen at her initial presentation

**Figure 3:**
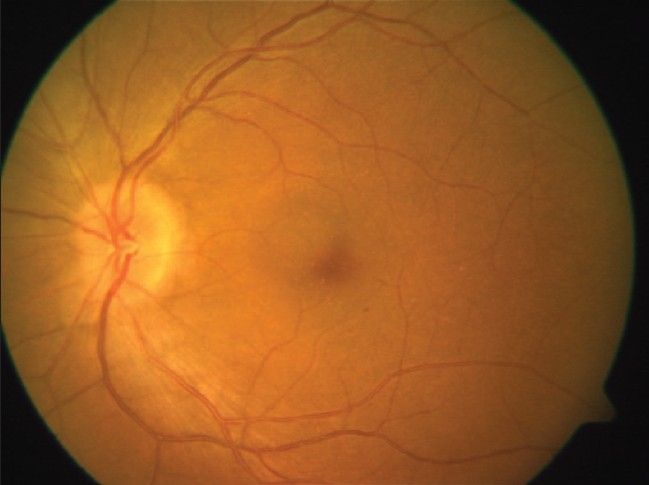
OS (Left eye) fundus photograph

**Figure 4:**
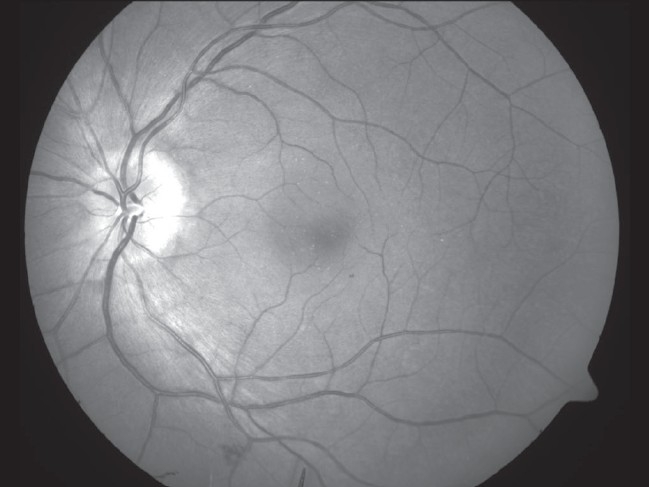
OS (Left eye) red free fundus photograph

**Figure 5:**
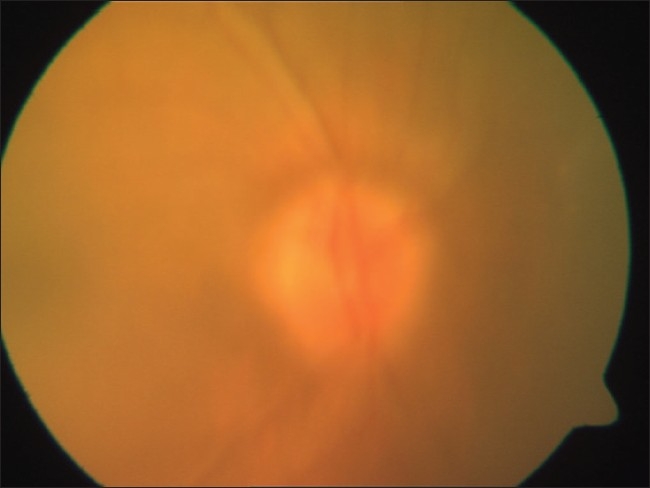
OD (Right eye) fundus photograph

**Figure 6:**
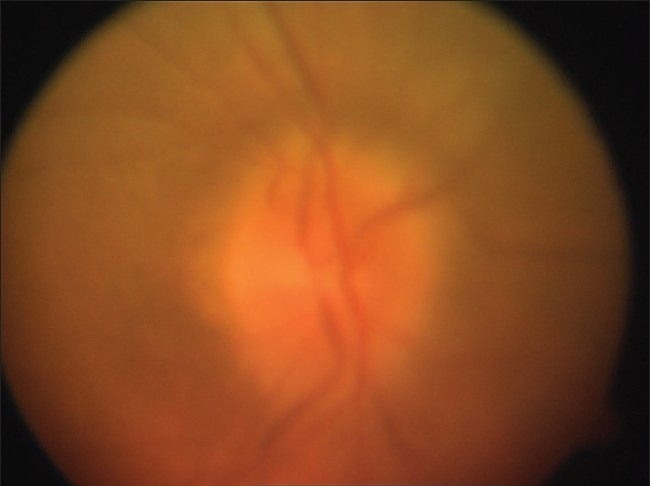
Photograph of the Right optic disc showing disc edema

**Figure 7:**
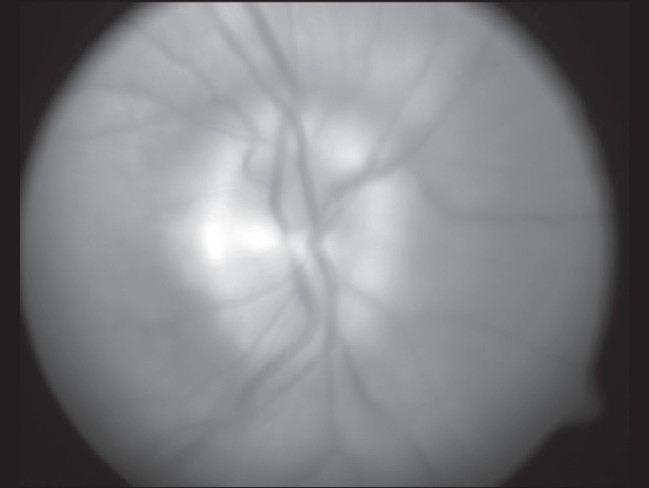
Red free photograph of the edematous Right optic disc.A central corneal scar affected clarity of the images of the Right eye

Optic disc edema can occur with or without a raised ICP. Cessation of the SVP is a sensitive marker of raised ICP. However, even in the presence of a normal ICP, optic disc edema has been considered to cause cessation of SVP.[2] We recently discovered pseudo-tumor cerebri in a 57-year-old female suffering from headache. Her vision was 20/40 in the right eye (RE), N8; counting fingers close to face in the left eye (LE) due to anisometropic amblyopia. SVP was seen on both edematous optic nerves in the erect posture and disappeared upon lying down (Figs 1–7). Magnetic resonance imaging revealed widened perioptic cerebrospinal fluid (CSF) spaces and empty sella. Her CSF opening pressure in lateral recumbent posture was 400 mm of H_2_O. She was not anemic and her systemic blood pressure was 140/90 mmHg.

SVP is seen in the majority of normal eyes in which the central retinal vein can be followed, unobscured by optic disc arteries or glial tissue, as it enters the cup.[3] However, the effect of optic disc size on the visibility of SVP has never been investigated. Logically, a large optic disc with a large physiologic cup would have more space to enable central vein to be seen entering the depth of the cup. In our case, optic discs were moderate to large (vertical diameter being 1.95 and 1.74 mm in RE and LE respectively) with a correspondingly larger physiological cup.

In pathological studies, a common pathway of axoplasmic stasis at the level of lamina cribrosa has been demonstrated to lead to optic disc edema of any etiology.[4] Moreover, in most individuals the amplitude of SVP is low and therefore the venous collapse spreads retrograde for a short distance from the exit point of the central retinal vein.[1] Therefore, when the ICP is normal, SVP may be obscured by the swollen axons but not necessarily diminished by them.

SVP is reported, albeit rarely, on a swollen optic disc in patients without elevated ICP.[2] On the other hand, McKee et al.[5] reported a case similar to ours. They could visualize SVP in the presence of a markedly elevated optic disc. The CSF opening pressure in their patient was 400 mm of H_2_O.[5] However, they have not commented on the size of the optic disc and the effect of posture on the visibility of SVP. A sudden lowering of the CSF pressure to normal does not produce immediate resolution of optic disc edema.[4] Owing to the effect of the spinal sac in CSF dynamics, a raised ICP is reduced in the erect posture. When such fluctuation in ICP touches normality, SVP may appear and be visible in a large edematous optic disc with a large physiologic cup as long as the optic cup is not completely obliterated by the swollen axons.
